# Do patients with metastatic pancreatic adenocarcinoma to the lung have improved survival?

**DOI:** 10.1002/cam4.5751

**Published:** 2023-03-14

**Authors:** Aren Ebrahimi, Jason Cham, Leah Puglisi, Melanie De Shadarevian, David J. Hermel, Samantha R. Spierling Bagsic, Darren Sigal

**Affiliations:** ^1^ Department of Internal Medicine Scripps Clinic and Scripps Green Hospital La Jolla California USA; ^2^ Scripps Whittier Diabetes Institute, Scripps Health San Diego California USA; ^3^ Department of Internal Medicine Scripps Mercy Hospital San Diego California USA; ^4^ Department of Hematology and Oncology Scripps Clinic and Scripps MD Anderson Cancer Center La Jolla California USA

**Keywords:** lung metastasis, pancreatic adenocarcinoma, survival

## Abstract

**Introduction:**

Pancreatic ductal adenocarcinoma (PDAC) is a genetically heterogeneous disease often diagnosed with synchronous metastatic disease involving the liver. Tumors with extra‐abdominal spread that bypass the liver are thought to represent a unique molecular subgroup and those with isolated pulmonary metastatic disease are thought to have a more favorable clinical phenotype.

**Method:**

We conducted a retrospective review of patients with pathologically confirmed PDAC treated between the years 2007 and 2020 at a Scripps Health hospital. The final study sample (*N* = 205) included patients with isolated pulmonary metastasis (IL), isolated liver metastasis or synchronous liver and lung metastasis (LL), or metastasis to any site other than the liver or lung (NLL). Primary endpoint was overall survival (OS). Progression‐free survival (PFS) and recurrence‐free survival (RFS) were analyzed as secondary endpoints. Each survival outcome was analyzed using Cox proportional hazards tests.

**Results:**

No statistically significant differences were seen between the three groups in OS, PFS, or RFS. Median OS for the IL group was 561 days, 341 days for the LL group, and 441 days for the NLL group. Median RFS was 748 days for the IL group, 574 days for the LL group, and 545 days for the NLL group. Median PFS was 307 for the IL group, 236 for the LL group, and 265 for the NLL group. When comparing only the IL and LL groups, a statistically significant difference in OS was seen favoring the IL group (HR1.59 LL vs IL [*ref*], CI 1.04–2.41, *p* = 0.031)

**Conclusion:**

Though statistically significant differences in survival outcomes were not seen in our population, there was a trend toward improved survival for patients with isolated lung metastases. When comparing only the IL to LL group, statistically significant overall survival favoring the IL group was seen. These findings highlight a potential prognostic indicator of metastatic PDAC.

## Graphical abstract

Overall survival, recurrence free survival and progression free survival outcomes of patients with pancreatic adenocarcinoma and different patterns of metastasis were compared. Patients with isolated lung metastasis showed favorable overall survival when compared to patients with isolated liver metastasis or synchronous liver and lung metastasis (HR 1.59, 95% CI 1.04–2.41; *p*‐value 0.031: reported as hazard ratio of death for the isolate liver or synchronous liver and lung group).

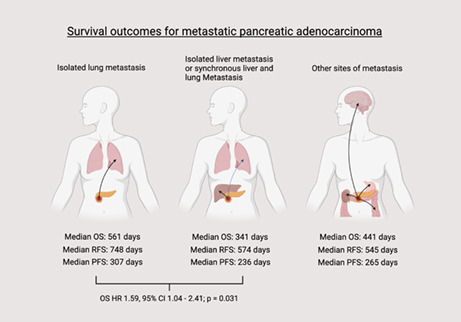

## 1 BACKGROUND

Pancreatic cancer remains a highly lethal malignancy with an estimated 49,830 deaths expected in the United States in 2022, representing 8.2% of all cancer‐related deaths and making it the third leading cause of cancer‐related death.^1^, ^2^ The national disease burden of pancreatic cancer is only expected to increase with projections estimating that it will become the second leading cause of cancer‐related death in the United States in the next twenty to thirty years.^1^ Despite advancements in our understanding of risk factors and improvements in early detection, the global burden of pancreatic cancer is projected to grow by an additional 350,000 new cases globally in 2040.^3^

Pancreatic ductal adenocarcinoma (PDAC) is the most common histologic subtype of pancreatic cancer representing over 90% of cases.^4^ PDAC is a genetically heterogeneous disease and patients often have advanced disease at the time of clinical presentation.^5^ Approximately 50% of patients will have metastases at time of diagnosis and another 30% of patients will have locally advanced and unresectable disease.^2^, ^3^ A minority of patients present with resectable disease, with surgery offering the only treatment with curative potential. Advances in adjuvant treatment have improved survival rates for resectable disease with overall 5‐year survival improved to 43% for resectable disease based on most recent SEERS data.^2^ Recent clinical trials have established systemic therapy with gemcitabine‐based multi‐drug therapy, often with nab‐paclitaxel, or modified folinic acid, fluorouracil, irinotecan, and oxaliplatin (mFOLFIRINOX) as standard adjuvant treatment.^6^, ^7^ Despite these advances in survival for select patients with resectable disease, 5‐year survival rates for distant metastasis remains at a dismal 3%.^2^

While the liver is the most common site of metastatic disease, recent insights into PDAC have elucidated unique clinical and molecular subgroups with distinct sites of extra‐abdominal spread.^8^, ^9^, ^10^, ^11^ For example, metastases that bypass the liver via portosystemic shunt more often arise from pancreatic body or tail tumors and have a unique genomic profile.^12^ Careful clinical investigation of patients with metastatic pancreatic cancer suggest that patients with isolated pulmonary recurrence of PDAC following surgery with curative intent have a more favorable clinical phenotype.^8^, ^13^, ^14^, ^15^ Following reports of this novel finding, we conducted a retrospective study to investigate whether patients with isolated pulmonary metastases, at time of recurrence or progression, had improved survival in metastatic PDAC.

## 2 METHODS

### 2.1 Study design

#### 2.1.1 Patient selection

**FIGURE 1:**
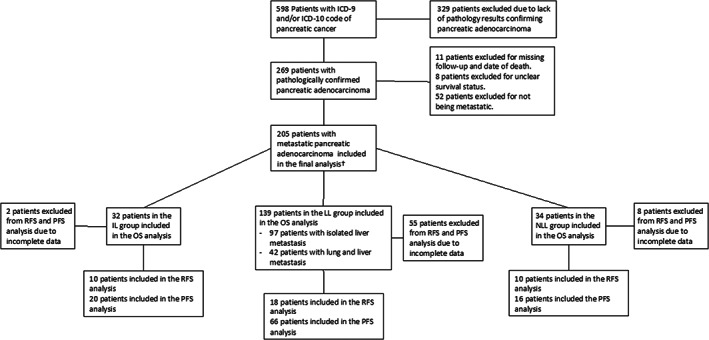
ICD, International Classification of Disease, IL, isolated lung metastasis group, LL, isolated liver metastasis or synchronous lung and liver metastasis, NLL, metastasis to site other than liver or lung, OS, overall survival, PFS, progression‐free survival, RFS, relapse‐free survival. ^†^Included patient who had metastasis noted at any time during study period. This would include patients noted to be metastatic at time of diagnosis, recurrence or progression.

We conducted a retrospective study of patients with a diagnosis of metastatic pancreatic cancer from May 1, 2007, to January 1, 2020 with a clinical encounter within our regional Scripps Health hospital system in San Diego County. The initial dataset considered for analysis consisted of 269 patients with pathology‐confirmed PDAC. Staging was determined based on the Union for International Cancer Control (UICC) Clinical TNM Staging criteria. The final sample size of patients analyzed was 205 (Figure 1). Patients were excluded from the study if follow‐up dates/death dates were not able to be appropriately identified (*N* = 11), if survival status was unclear (*N* = 8), or if there were no sites of metastases identified at time of diagnosis or recurrence/progression (*N* = 52). Patients were then categorized into isolated lung metastases (IL), isolated liver metastases or synchronous lung and liver metastases (LL), and metastasis other than the liver or lung (NLL). Presence of both lung and liver metastasis was grouped together with isolated liver metastasis in survival analysis given synchronous metastasis would likely involve hematogenous spread first through the portal system. Patients were also categorized into recurrence‐free survival and progression‐free survival cohorts. Recurrence‐free survival was defined as the time from diagnosis to first imaging evidence of recurrence in patients who received surgery with curative intent (pancreatoduodenectomy, distal pancreatectomy or total pancreatectomy). Progression‐free survival was defined as the time from diagnosis to first imaging evidence of progression of disease in patients who did not receive potentially curative surgery. Imaging modalities reviewed for recurrence or progression included computer tomography (CT), magnetic resonance imaging (MRI), or positron emission tomography (PET)/CT. This study was reviewed and approved by the Scripps Health Institutional Review Board (IRB): IRB‐19‐7463.

#### 2.1.2 Data collection

A total of 598 patient charts were initially identified with an ICD‐9 or ICD‐10 diagnosis of pancreatic cancer. Following confirmation of diagnosis of cancer by pathology, retrospective data on demographic and clinical information was collected into the de‐identified centralized database. Additional data on demographics, pre‐existing medical co‐morbidities, CA 19–9 at time of diagnosis, date of initial diagnosis of pancreatic cancer by pathological confirmation, pathology results from tumor biopsy or surgical pathology following potentially curative surgery, date of recurrence as evident on imaging, date of progression as evident on imaging, metastasis as evident on imaging, treatments received, date of most recent follow‐up, survival status, and date of death were collected.

#### 2.1.3 Statistical analyses

Demographic data were summarized as the number of occurrences and percentages for categorical data, and as means and standard deviations (SD) for continuous data. Baseline characteristics were compared among the three groups of metastases (IL, LL, and NLL) by either a chi‐square test for categorical data or one‐way analysis of variance (ANOVA) for continuous variables. Possible clinical variables associated with isolated lung metastases versus lung and liver metastases among PDAC patients were examined by univariable and multivariable logistic regressions. Survival time was analyzed using the Kaplan–Meier method to understand survival among the 3 groups of metastases (IL, LL, and NLL). Log‐rank tests were used to determine the significance of differences between the survival curves. To describe how the factors in our study jointly impact survival, univariable and multivariable analyses using unadjusted Cox proportional hazard models were run for the outcomes of overall patient survival, recurrence‐free survival, and progression‐free survival, and we report hazard ratios (HRs) and their confidence intervals (CIs). This was to investigate the relationship between time to event (death or recurrence/progression) and a set of explanatory variables. All the variables in univariable analysis with *p*‐values <0.05 were further analyzed using a multivariable analysis. All *p‐*values were 2‐tailed, and *p‐*values <0.05 were considered statistically significant. In the confidence intervals reported, not available (NA) values were used when there was not enough people who had the corresponding event. The full multivariable models for each survival outcome included the variables identified as being significant in the univarible analyses and included those with complete data. The statistical software R (v.4.1.3) was used for all analyses and figure generation.

## 3 RESULTS

A total of *N* = 205 patients with PDAC were identified during the study period. There were 32 patients with isolated lung metastases, 97 with isolated liver metastases, 42 had lung and liver metastases, and 34 had any other metastasis other than lung or liver metastases. 38 patients had received surgery with curative intent and were categorized into the RFS group. The remaining 167 patients were categorized into the PFS group. Figure 1 illustrates patient selection and grouping for this study. Overall baseline characteristics of the study population are shown in Table 1 **.** The mean age of the patients was 69 ± 9.3 years old, with the majority being male (57.1%) and white (78%). 51.2% of patients had 1 metastatic organ with the most common metastatic organ being liver (48.8% at time of diagnosis, 39.5% at time of recurrence or progression), followed by lung (17.1% at time of diagnosis, 26.3% at time recurrence or progression). Mortality during the study period was 83.4%.

**TABLE 1:** Baseline characteristics between IL, LL, and NLL groups.

Variable	Factor	IL (N = 32)	LL (N = 139)	NLL (N = 34)	*p*‐value
Age	Mean ± SD [range]	69 ± 9.3 [52, 84]	68.2 ± 11 [38, 94]	70.2 ± 9.2 [50, 88]	0.599
Sex	Male	17 (53.1%)	84 (60.4%)	16 (47.2%)	0.327
	Female	15 (46.9%)	55 (39.6%)	18 (52.9%)	
Ethnicity	Hispanic	4 (12.5%)	12 (8.6%)	3 (8.8%)	0.745
	White	22 (68.8%)	111 (79.9%)	27 (79.4%)	
	Other	6 (18.8%)	16 (11.5%)	4 (11.8%)	
CA 19–9	≤37	5 (15.6%)	13 (9.4%)	2 (5.9%)	0.168
	>37	17 (53.1%)	108 (77.7%)	29 (85.3%)	
	Missing	10 (31.3%)	18 (12.9%)	5 (8.8%)	
Alcohol	Prior/Current	19 (59.4%)	82 (59.0%)	22 (64.7%)	0.546
	Never	12 (37.5%)	47 (33.8%)	8 (23.5%)	
	Missing	1 (3.1%)	10 (7.2%)	4 (11.8%)	
Smoking	Prior/Current	12 (37.5%)	54 (38.8%)	12 (35.3%)	0.954
	Never	19 (59.4%)	76 (54.7%)	18 (52.9%)	
	Missing	1 (3.1%)	9 (6.5%)	4 (11.8%)	
Medical History	CHF	1 (3.1%)	5 (3.6%)	2 (5.9%)	0.802
	CAD	6 (18.8%)	19 (13.7%)	4 (11.8%)	0.690
	HLD	17 (53.1%)	48 (34.5%)	6 (17.6%)	0.010
	HTN	18 (56.3%)	68 (48.9%)	16 (47.1%)	0.713
	Cirrhosis	3 (9.4%)	0	0	NA
	Hepatitis B	0	1 (0.7%)	0	NA
	Hepatitis C	1 (3.1%)	4 (2.9%)	0	NA
	Pancreatic Cyst	1 (3.1%)	3 (2.2%)	2 (5.9%)	0.512
	Anemia	4 (12.5%)	15 (10.8%)	4 (11.8%)	0.957
	Diabetes Mellitus	7 (21.9%)	25 (18.0%)	13 (38.2%)	0.038
	Hypothyroidism	4 (12.5%)	17 (12.2%)	4 (11.8%)	0.996
	CKD	2 (6.3%)	8 (5.8%)	4 (11.8%)	0.456
	None	4 (12.5%)	31 (22.3%)	7 (20.6%)	0.464
Stage	1	2 (6.3%)	6 (4.3%)	4 (11.8%)	<0.001
	2	9 (28.1%)	18 (12.9%)	17 (50.0%)	
	3	7 (21.9%)	12 (8.6%)	4 (11.8%)	
	4	12 (37.5%)	94 (67.6%)	7 (20.6%)	
	Missing	2 (6.3%)	9 (6.5%)	2 (5.9%)	
Resectability	Resectable	7 (21.9%)	19 (13.7%)	9 (26.5%)	<0.001
	Locally Advanced	10 (31.3%)	23 (16.5%)	19 (55.9%)	
	Metastatic	14 (43.8%)	96 (69.1%)	6 (17.6%)	
	Missing	1 (3.1%)	1 (0.7%)	0	
Line	None	4 (12.5%)	27 (19.4%)	6 (17.6%)	0.009
	1st	3 (9.4%)	46 (33.1%)	9 (26.5%)	
	2nd	3 (9.4%)	14 (10.1%)	8 (23.5%)	
	3rd	22 (68.8%)	52 (37.4%)	11 (32.4%)	
Initial Surgery	Yes	11 (34.4%)	21 (15.1%)	11 (32.4%)	0.014
	No	21 (65.6%)	115 (82.7%)	23 (67.6%)	
	Missing	0	3 (2.2%)	0	
Initial Radiation	Yes	13 (40.6%)	24 (17.3%)	9 (26.5%)	0.014
	No	18 (56.3%)	111 (79.9%)	25 (73.5%)	
	Missing	1 (3.1%)	4 (2.9%)	0	
Recurrent disease Radiation therapy	Yes	8 (25.0%)	14 (10.1%)	12 (35.3%)	0.005
	No	21 (65.56%)	97 (69.8%)	21 (61.8%)	
	Missing	3 (9.4%)	28 (20.1%)	1 (2.9%)	
Recurrent Disease Surgery	Yes	0	3 (2.2%)	2 (5.9%)	0.368
	No	29 (90.6%)	110 (79.1%)	32 (94.1%)	
	Missing	3 (9.4%)	26 (18.7%)	0	
Recurrent Disease Adjuvant Therapy	Yes	7 (21.9%)	16 (11.5%)	9 (26.5%)	0.171
	No	18 (56.3%)	90 (64.7%)	25 (73.5%)	
	Missing	7 (21.9%)	33 (23.7%)	0	

*Note*: Values are presented as total number and percentage of variable within each group. *p*‐values <0.05 were considered statistically significant. If information on a variable was not available for a patient, this was categorized as “missing.” “NA” in the confidence intervals were written when there were not enough people who had the corresponding estimate the upper limit of the confidence interval.

Abbreviations: CAD, coronary artery disease; CKD, chronic kidney disease; CHF, congestive heart failure; HLD, hyperlipidemia; HTN, hypertension.

Comparison of baseline characteristics for the three groups of metastases is shown in Table 1. There was no statistically significant difference in age, sex, ethnicity, smoking, and alcohol use, history of pancreatic cyst, or CA 19–9 at time of diagnosis seen between the three groups. A statistically significant difference in diabetes mellitus as a comorbidity was noted (*p* = 0.038) with 21.9% in the IL group, 18.0% in the LL group, and 38.2% in the NLL group. Complete results for demographic variables, tumor variables, treatments, and frequency of sites of metastasis can be seen in Tables S1–S5.

**FIGURE 2:**
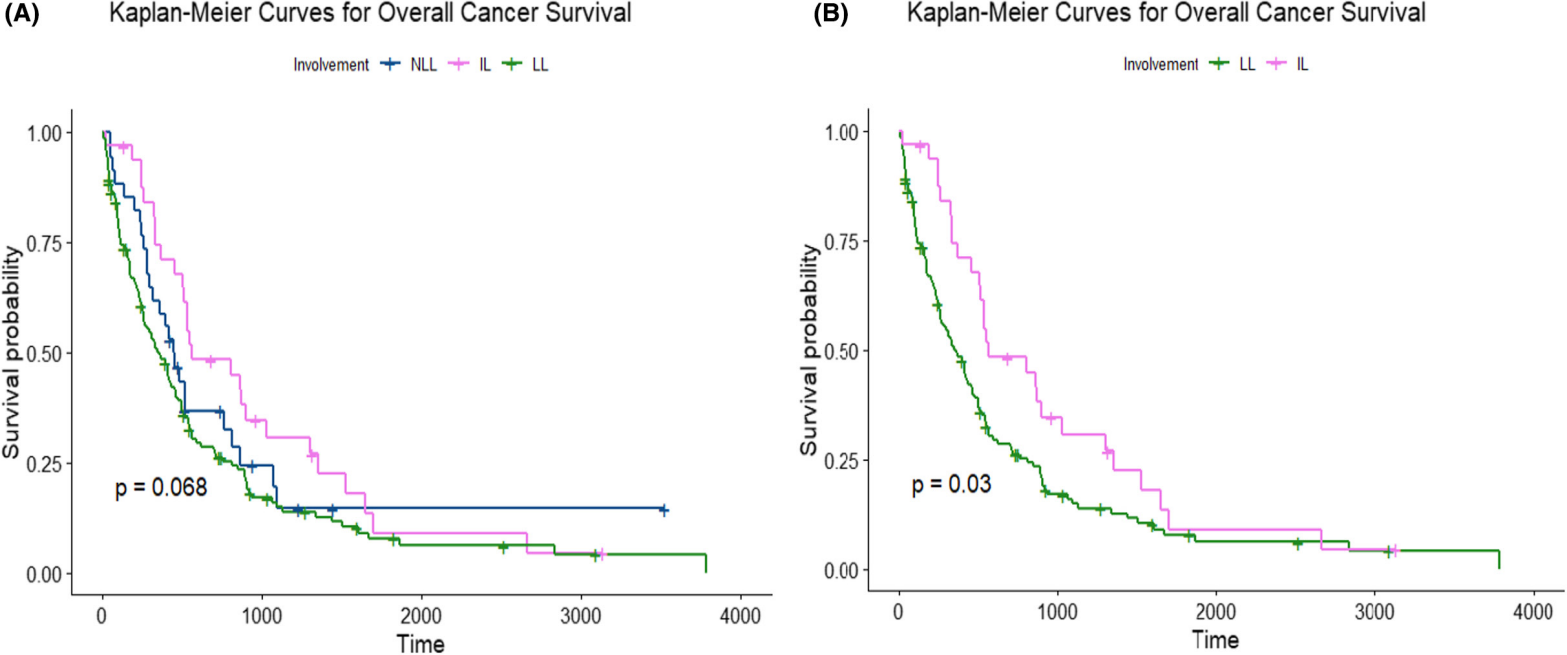
Kaplan–Meier survival curves for overall survival for (A) all three groups and (B) when comparing only IL to LL. IL, isolated lung metastases. LL, isolated liver metastases or synchronous lung and liver metastases. NLL, metastasis other than the liver or lung.

The overall median survival time for the entire study cohort (*N* = 205) was 417 days (95% CI 331–501). As shown in Table 2 **,** median survival time of the IL group was 561 days (95% CI 501–1301), median survival time of the LL group was 341 days (95%, CI 255–455), and median survival time of the NLL group was 441 days (95% CI 317–861). There was no statistical difference in overall survival outcome between these 3 groups (Tables 3, Figure 2). As shown in Table 4, when comparing IL to LL, a statistically significant difference in survival was seen favoring the IL group (HR 1.59, 95% CI 1.04–2.41; *p*‐value 0.031: reported as hazard ratio of death for the LL group). Kaplan–Meier Survival Curve for overall survival of IL compared to LL can be seen in Figure 2.

**TABLE 2:** Outcomes for overall survival, recurrence‐free survival and progression‐free survival for the IL, LL, and NLL groups.

	N	Events	Median (days)	95%CI (days)
Overall survival^a^				
NLL	34	26	441	317, 861
IL	32	27	561	501, 1301
LL	139	118	341	255, 455
Recurrence free survival^b^				
NLL	10	10	545	337, NA
IL	10	10	748	394, NA
LL	18	18	574	553, 1322
Progression free survival^c^				
NLL	16	16	265	172, 470
IL	20	19	307	250, 419
LL	66	66	236	197, 302

*Note*: “NA” in the confidence intervals were written when there were not enough people who had the corresponding estimate the upper limit of the confidence interval.

^a^ Overall survival: N = 205, number of events = 171 with event defined as death.

^b^ Recurrence‐free survival: N = 38, number of events = 38 with event defined as recurrence.

^c^ Progression‐free survival: N = 102, number of events = 101 with event defined as progression.

**TABLE 3:** Survival analysis for overall survival, recurrence‐free survival, and progression‐free survival among all three groups (IL, LL, and NLL).

	HR	95%CI	*p*‐value
Overall survival			
NLL	Ref	Ref	Ref
IL	0.79	(0.46, 1.36)	0.3920
LL	1.26	(0.83, 1.94)	0.2800
Recurrence‐free survival			
NLL	Ref	Ref	Ref
IL	0.52	(0.21, 1.29)	0.158
LL	0.65	(0.29,1.46)	0.297
Progression‐free survival			
NLL	Ref	Ref	Ref
IL	0.88	(0.45, 1.71)	0.698
LL	1.00	(0.58, 1.75)	0.976

*Note*: CI, confidence interval, HR, hazard ratio. HR >1 would denote a greater probability of the corresponding event and <1 would denote a lower probability of the corresponding event (death, recurrence, or progression).

**TABLE 4:** Survival analysis for overall survival, recurrence‐free survival, and progression with only IL and LL groups.

	HR	95%CI	*p*‐value
Overall survival			
IL	Ref	Ref	Ref
LL	1.59	1.04, 2.41	0.031
Recurrence free survival			
IL	Ref	Ref	Ref
LL	1.25	0.57, 2.76	0.577
Progression free survival			
IL	Ref	Ref	Ref
LL	1.16	0.70, 1.95	0.562

*Note*: CI, confidence interval, HR, hazard ratio. HR >1 would denote a greater probability of the corresponding event and <1 would denote a lower probability of the corresponding event (death, recurrence or progression).

The overall median survival time in the RFS cohort (*N* = 38) was 1348 days (95% CI 917–2664). Median survival for the IL group in the RFS cohort was 1650 days (95% CI 1301 ‐ NA), median survival for the LL group in the RFS cohort was 1503 days (95% CI 903 ‐ NA), and median survival for the NLL group in the RFS cohort was 1091 days (95% CI 514 ‐ NA) (Table S6). There was no statistical difference in median survival between these 3 groups (Table S7, Figure S1). When comparing the IL group to the LL group in the RFS cohort, the difference in overall survival was not statistically significant (HR 1.30, 95% CI 0.51–3.34) (Table S8, Figure S1). Overall median time to recurrence was 595 days (95% CI 553–804). As shown in Table 2 **,** the IL group had a median time to recurrence of 748 days (95% CI 394 ‐ NA), the LL group had a median time to recurrence of 574 days (95% CI 553–1322), and the NLL group had a median time to recurrence of 545 days (95% CI 337 ‐ NA). There was no statistical difference in time to recurrence between these 3 groups or when comparing just the IL group to LL group (Tables 3 and 4 **).** Kaplan–Meier survival curve for time to recurrence can be seen in Figure S2.

The overall median survival time in the PFS cohort was 322 days (95% CI 256–407). The IL group in the PFS cohort had a median survival time of 501 days (95% CI 330–804), the LL group had a median survival time of 275 days (95% CI 225–406), and the NLL group had a median survival time of 304 days (95% CI 255–513; Table S6). There was no statistical difference in median survival between these 3 groups or when comparing the IL group to the LL group (Tables S7, S8, Figure S3). The overall median time to progression in the PFS group was 250 days (95% CI 224–302). As shown in Table 2, the IL group had a median time to progression of 307 days (95% CI 250–419), the LL group had a median time to progression of 236 days (95% CI 197–302), and the NLL group had a median time to progression of 265 days (95% CI 172–470). There was no statistical difference in time to progression between these 3 groups or when comparing just the IL group to the LL group (Tables 3, 4). Kaplan–Meier survival curve for time to progression can be seen in Figure S4.

Finally, logistical regression analysis was performed to identify predictors of lung or liver metastasis. Two variables were identified as being statistically significantly in univariable analysis; having a medical history of hyperlipidemia (OR 2.30 for lung metastasis, 95% CI 1.02–5.25; *p* = 0.0445) and moderately differentiated tumor (OR 0.05 for lung metastasis, 95% CI 0.00–0.60; *p* = 0.0236). Moderately differentiated tumors remained an independent risk factor associated with decreased probability of lung metastases in the multivariable analysis (Table 5).

**TABLE 5:** Logistic regression analysis for clinical factors associated with predicting lung or liver involvement.

			Univariate		Multivariate	
Variable	Factor	N/%	OR (95%CI)	*p*‐value	OR (95%CI)	*p*‐value
Sex	Male	76 (58.9%)	Ref	Ref		
	Female	53 (41.1%)	1.37 (0.61, 3.07)	0.44		
Age	Mean ± SD [Range]	67.74 ± 10.67 [38, 94]	1.01 (0.97,1.05)	0.522		
Ethnicity	White	98 (76%)	Ref	Ref		
	Hispanic	12 (9.3%)	1.73 (0.43, 6.05)	0.407		
	Other	19 (14.7%)	1.60 (0.51, 4.56)	0.396		
CA19‐9	≤37	16 (14.7%)	Ref	Ref		
	>37	93 (85.3%)	0.49 (0.16, 1.73)	0.239		
Smoker	Never	70 (57.9%)	Ref	Ref		
	Current/Prior	51 (42.1%)	0.83 (0.35, 1.89)	0.6532		
Alcohol	Never	40 (33.1%)	Ref	Ref		
	Current/Prior	81 (66.9%)	0.72 (0.31, 1.70)	0.4390		
Diabetes	No	104 (80.6%)	Ref	Ref		
	Yes	25 (19.4%)	1.23 (0.44, 3.19)	0.681		
Pancreatic cyst	No	126 (97.7%)	Ref	Ref		
	Yes	3 (2.3%)	1.53 (0.07, 16.53)	0.731		
Hyperlipidemia	No	80 (62%)	Ref	Ref	Ref	Ref
	Yes	49 (38%)	2.30 (1.02, 5.25)	0.0445	1.44 (0.47, 4.38)	0.5191
Invasion^a^	No	24 (21.1%)	Ref	Ref		
	Yes	90 (78.9%)	2.14 (0.73, 7.89)	0.1995		
Tumor location^a^	Head	80 (63.0%)	Ref	Ref		
	Body and Tail	45 (35.4%)	1.25 (0.53, 2.90)	0.601		
	Diffuse	2 (1.6%)	5.98 (NA, INF)	0.989		
Tumor differentiation^a^	Highly Differentiated	3 (3.3%)	Ref	Ref	Ref	Ref
	Undifferentiated or Poorly	51 (56.7%)	0.19 (0.01, 2.12)	0.1879	0.23 (0.01, 2.76)	0.2595
	Moderately	36 (40%)	0.05 (0.00, 0.60)	0.0236	0.06 (0.00, 0.85)	0.0448
Lymph node status^a^	Unknown	70 (54.3%)	Ref	Ref		
	0	17 (13.2%)	0.21 (0.01, 1.16)	0.1456		
	1–3	24 (18.6%)	2.4 (0.89, 6.48)	0.0799		
	≥4	18 (14.0%)	1.30 (0.37, 4.04)	0.6628		
Resectability^a^	Resectable	23 (18.1%)	Ref	Ref		
	Locally Advanced	25 (19.7%)	1.52 (0.46, 5.20)	0.4898		
	Metastatic	79 (62.3%)	0.49 (0.17, 1.48)	0.1898		
Tumor stage^a^	1	7 (5.7%)	Ref	Ref		
	2	21 (17.2%)	1.88 (0.32, 15.25)	0.506		
	3	18 (14.0%)	1.60 (0.26, 13.32)	0.631		
	4	76 (62.3%)	0.47 (0.09, 3.53)	0.397		

*Note*: All the variables in univariable analysis with *p*‐values <0.05 were considered statistically significant and were further analyzed using a multivariable analysis. OR, odds ratio. OR >1 would denote greater probability of predicting lung metastasis and < 1 would denote lower probability of predicting lung involvement.

^a^ These variables reported above are tumor characteristic from initial pathology and imaging findings. Invasion refers to vascular and/or perineural invasion.

## 4 DISCUSSION

In our study, no significant difference in overall survival was observed when comparing outcomes among patients with PDAC stratified into three separate groups based on associated lung and/or liver tumor involvement. Though there was no benefit in OS, PFS, and RFS among these three groups, a statistically significant survival benefit was noted in the IL group compared with the LL group in the overall survival cohort.

This finding is consistent with favorable outcomes for isolated lung metastasis when compared to liver metastasis seen in other studies. One large meta‐analysis evaluated the survival outcomes for PDAC with unresected metastasis or recurrence isolated to the lung as compared to other sites.^8^ This analysis included 15 studies, of which one was an RCT and one an analysis of the SEER database, with an aggregate of 11,916 patients, 1199 of which had isolated pulmonary metastasis. When compared to liver metastasis, they noted a mean difference of 10.89 months (95% CI 3.81–17.98, *p* < 0.0001; I^2^ 6%) in favor of pulmonary metastasis for overall survival. Similarly, they noted improved survival of pulmonary metastasis vs locoregional recurrence with mean difference of 9.25 months (95% CI 3.42–15.08, *p* = 0.002; I^2^ 0%). The weighted median OS of patients with isolated lung metastasis was 14.6 months (1085 patients), with pulmonary recurrence after pancreatectomy having median OS of 34.7 months (286 patients) and unresected PDAC with isolated lung metastasis having median OS of 7.3 months (799 patients).^8^ In our study, the median OS for patients in the IL group was 18.4 months (561 days) with 54.3 months (1650 days) for the RFS cohort and 16.5 months (501 days) for the PFS cohort. In comparison, the weight median OS for hepatic metastasis was 11.3 months in the meta‐analysis and 11.2 (341 days) months in our study.

We did not identify a statistically significant difference in survival or time to recurrence in the RFS cohort. This cohort included patients who received surgery with curative intent, with or without neoadjuvant chemotherapy. Recent studies investigating survival outcomes with patterns of recurrence in PDAC have found statistically significant favorability for isolated and primary lung metastasis.^8^, ^13^, ^14^, ^15^ PDAC patients who underwent surgery with curative intent and had subsequent metastasis to the lung as first location of recurrence achieved longer disease‐free survival and survival after recurrence as compared to patient with locoregional relapse or disease progression in the liver or peritoneum.^8^ In their systematic review and meta‐analysis, Tanaka et al. also sought to better understand clinicopathological features and clinical outcomes associated with recurrence patterns for PDAC following surgery with curative intent.^14^ The authors identified survival trends favoring lung recurrence when compared to locoregional recurrence, liver recurrence or peritoneal dissemination. Median OS for lung recurrence was 30.4 months, median RFS was 15.6 months and survival after recurrence was 12.1 months. Median OS, median RFS and survival after recurrence was 15.0, 7.7 and 6.5 months for patients with liver recurrence, 15.0, 12.7, and 7.9 months for patients with locoregional recurrence, and 14.1, 8.8, and 4.4 months for patients with peritoneal dissemination.^14^ Finally, a retrospective study by Sahin et al. investigated whether time from relapse to death differed for PDAC patients having recurrence in the lung compared to liver following surgery with curative intent. In their single‐center study, the authors identified 149 eligible patients over a 10‐year period, 102 of which had recurrence in the liver and 47 with recurrence in the lung. Median time from relapse to death for the overall cohort was 10.7 months (95% CI 8.9–14.6) with a statistically significant (*p* = 0.02) difference favoring the lung metastasis group (15 months, 95% CI 11–18) over the liver metastasis group (9 months, 95% CI 7–11).^13^ Despite both OS and RFS trending in favor of lung metastasis in this cohort of our study, statistical significance was not seen. This is likely due to the smaller sample sizes in our study.

Similarly, median OS and time to progression in the PFS cohort in our study did not reach statistical significance despite a trend in favor of lung metastasis. This cohort included patients who did not receive surgery with curative intent and would have received palliative chemotherapy or no treatment. In their retrospective study, Liu et al. looked at survival outcomes for stage IV pancreatic cancer patients receiving palliative chemotherapy with different patterns of metastasis.^16^ The authors identified 746 patients in four medical centers in Taiwan over a 7‐year study period (2010–2016). In their study, the isolated lung group, which included 3.4% of the study population, had a median OS of 11.8 months (95% CI, 6.7–17.0). The rest of their study population was distributed into isolated liver, isolated peritoneum, isolated distant lymph nodes, and multiple sites of metastasis. Median OS for these other groups was 6.9 (95% CI, 5.8–8.0), 7.7 (95% CI, 6.4–9.1), 10.1 (95% CI, 8.0–12.2), and 5.0 (95% CI, 4.3–5.7) months, respectively. The isolated lung metastasis group had improved median OS that was statistically significant in comparison with isolated liver (*p* = 0.005) and multiple sites of metastasis (*p* < 0.001). Despite longer survival times, survival outcomes did not reach statistical significance when compared to the other two groups in this study.^16^

Isolated lung metastasis was detected in 32 (15.6%) of 205 patients in our study population. When compared to liver metastasis, univariable analysis demonstrated lower probability of isolated lung metastasis for patients with moderately differentiated tumors and higher probability for patients with hyperlipidemia. Moderately differentiated tumors remained an independent risk factor in the multivariable analysis. In their univariable analysis, Liu et al identified female gender (OR 1.88 [95% CI 1.22–2.89], *p* = 0.004), poorly differentiated or undifferentiated tumor grade (OR 3.83 [95% CI 1.18–12.5], *p* = 0.026), and primary tumor size > = 8 cm (OR 2.21 [95 CI% 1.10–4.45], *p* = 0.026) as factors associated with higher probability of lung metastasis. All three remained as independent factors associated with increased probability of lung metastasis in multivariable analysis.^16^ Sahin et al. also investigated predictors of liver or lung metastasis in their retrospective study and found that in the liver metastasis cohort, there was significantly more moderate‐poor or poorly differentiated PDAC's patients (*p* = 0.047) and patients with higher BMI (*p* = 0.05). Of note, the authors had moderately differentiated as a separate category than moderate‐poor or poorly differentiated, and the lung metastasis group had a higher percentage of moderate differentiation (77% vs. 59%).^13^ Finally, in their meta‐analysis, Tanaka et al. also reviewed histopathological differences as predictors of sites of metastasis and found that moderate and poor differentiation was significantly associated with liver recurrence (OR 4.15).^14^ As tumors become less differentiated, they have more aggressive biology leading to higher risk of metastasis. Specific genes and pathways, such as FOXA1 and Sonic hedgehog (SHH), have been shown to contribute to tumor metastasis.^17^ However, the exact molecular alterations leading to the associated metastatic potential remains unclear.

Our retrospective study is unique in that it investigates survival outcomes for both patients with recurrence following curative surgery and patients who had only received palliative treatment from the same overall cohort. To the best of our knowledge, other individual studies investigating survival outcomes for lung metastasis in PDAC, compared to other sites of metastasis, had looked at either recurrence following surgery or progression with palliative treatment. The small sample size for RFS and PFS cohorts individually is a limitation of the current study. Furthermore, in our study, we have grouped all non‐liver and non‐lung metastasis into the NLL group. This was done to simplify the comparative groups and due to small sample sizes of isolated peritoneal, locoregional or other metastasis outside the liver or lung. However, as noted above from other studies, these patterns of recurrence and metastasis also likely have unique prognostic outcomes. Finally, only a few patients in our cohort had pathology confirmation of metastatic PDAC from biopsy taken from the site of metastasis. Using the initial imaging evidence of metastasis may have incorrectly categorized patients into the isolated lung or isolated liver groups who had synchronous metastasis to multiple sites near the time of initial imaging but with initial imaging demonstrating only isolated metastasis.

Despite these limitations, the findings of this study add to a growing body of literature showing favorable outcomes for patients with isolated or primary metastasis to the lung.^8^, ^13^, ^14^, ^15^, ^16^, ^18^ Understanding the prognostic differences associated with unique patterns of metastasis in PDAC can help guide treatment plans and help stratify patients for clinical trials. For example, metastasis in PDAC often precludes patients from surgical intervention, however, given significant survival differences seen with isolated lung metastasis, surgical resection of lung metastasis can be a potentially promising treatment choice with improved survival and less toxicity than palliative chemotherapy.^14^, ^19^, ^20^, ^21^, ^22^, ^23^ Furthermore, the trend toward earlier progression and recurrence for liver metastasis seen in our study and others can help support decisions for pursuing more aggressive systemic chemotherapy in select patients.^13^, ^16^, ^18^ At this time, guideline‐based treatment to metastatic pancreatic cancer has an equivocal approach without regard to prognostic differences associated with unique patterns of metastasis.^24^ Tumor genetics and histopathological characteristics will also undoubtedly play a role going forward. PDAC is a genetically heterogeneous disease with varied responses to systemic chemotherapy and targeted therapies.^5^, ^9^ Other studies have begun to highlight the unique biological differences associated with different patterns of metastasis in PDAC and tumor aggression.^5^, ^8^, ^9^, ^10^, ^11^ Unique subclonal cell populations within the primary tumor harbor the unique profile of genetic alterations and chromosomal instability seen in metastasis. This heterogeneity within subclonal cell populations leads to heterogeneity seen between distinct sites of metastasis, where further superimposed mutations occur.^5^ As an example, KRAS and PTEN mutations have been seen at greater proportion in metastasis to the lung, highlighting these as potential predictors of metastatic or recurrence patterns.^8^, ^10^, ^11^ At this current time however, further research into the biological diversity of PDAC is needed to better understand the potentially targetable mutations and to predict tumor behavior for patient stratification into clinical trials and therapy choices following metastasis.^8^, ^25^

## 5 CONCLUSION

In summary, we observed a statistically significant overall survival difference in favor of isolated and initial lung metastasis compared to liver metastasis. We also observed a trend toward improved overall survival for lung metastasis when compared to other sites of metastasis in PDAC, as well as a trend toward longer RFS and PFS. Unique clinicopathologic features were associated with lung metastasis in comparison with liver metastasis and there is growing body of evidence supporting individualized approaches to patient care owing to the variation in clinical features associated with unique patterns of metastasis and recurrence. Further prospective studies are needed to understand clinical outcomes associated with varied approaches to treatment of PDAC following recurrence and metastasis.

## AUTHOR CONTRIBUTIONS

**Aren Ebrahimi:** Data curation (equal); investigation (equal); methodology (equal); writing – original draft (lead); writing – review and editing (lead). **Jason Cham:** Conceptualization (equal); data curation (equal); funding acquisition (equal); investigation (equal); methodology (equal); writing – original draft (supporting); writing – review and editing (supporting). **Leah Puglisi:** Formal analysis (equal); methodology (equal); writing – original draft (supporting); writing – review and editing (supporting). **Melanie De Shadarevian:** Investigation (supporting); writing – original draft (supporting). **David Hermel:** Conceptualization (equal); methodology (equal); supervision (equal); writing – original draft (supporting); writing – review and editing (supporting). **Samantha Bagsic:** Data curation (equal); formal analysis (equal); methodology (equal); writing – original draft (supporting). **Darren Sigal:** Conceptualization (lead); investigation (equal); methodology (equal); project administration (lead); supervision (lead); validation (lead); writing – original draft (supporting); writing – review and editing (supporting).

## FUNDING INFORMATION

JC is supported by the linked award KL2TR002552. The content is solely the responsibility of the authors and does not necessarily represent the official views of the NIH.

## CONFLICT OF INTEREST STATEMENT

None reported. All authors have declared there are no financial conflicts of interest with regard to this work.

## ETHICS APPROVAL AND PATIENT CONSENT TO PARTICIPATE

IRB approval obtained through Scripps Health IRB: IRB‐19‐7463.

## Supporting information


Data S1:

## Data Availability

All data used to support the findings above are available upon reasonable request.
