# Supplementary material for: Do patients with metastatic pancreatic adenocarcinoma to the lung have improved survival?
Source: Cancer Med. 2023 Mar 14;12(9):10243–53. doi: 10.1002/cam4.5751 (PMC10225201; doi:10.1002/cam4.5751)
Supplement: Supplementary file 1 — Data S1: [file CAM4-12-10243-s001.docx]

**Supplemental Table 1: Demographic variables for entire study cohort**

|  | N=205 |  |  |
| --- | --- | --- | --- |
| Age mean ± SD [range] | 68.6 ± 10.45 | [38,94] |  |
|  | **Factor/Category** | **Frequency** | **Percent** |
| Subgroup | RFS | 38 | 18.5% |
|  | PFS | 167 | 81.5% |
| Sex | *Male* | 117 | 57.1% |
|  | *Female* | 88 | 42.9% |
| Ethnicity | *White* | 160 | 78.0% |
|  | *Hispanic/Latino* | 19 | 9.3% |
|  | *Other* | 26 | 12.7% |
| Medical History | *CHF* | 8 | 3.9% |
|  | *CAD* | 29 | 14.1% |
|  | *HLD* | 71 | 34.6% |
|  | *HTN* | 102 | 49.8% |
|  | *Cirrhosis* | 3 | 1.5% |
|  | *Hep B* | 1 | 0.5% |
|  | *Hep C* | 5 | 2.4% |
|  | *Pancreatic Cyst* | 6 | 2.9% |
|  | *Anemia* | 23 | 11.2% |
|  | *Diabetes Mellitus* | 45 | 22.0% |
|  | *Hypothyroidism* | 25 | 12.2% |
|  | *CKD* | 14 | 6.8% |
|  | *None* | 42 | 20.5% |
| CA19-9 | *≤37* | 20 | 9.8% |
|  | *>37* | 154 | 75.1% |
|  | *Missing* | 31 | 84.9% |
| Alcohol | *Prior/Current* | 123 | 60% |
|  | *Never* | 67 | 32.7% |
|  | *Missing* | 15 | 7.3% |
| Smoking | *Prior/Current* | 78 | 38% |
|  | *Never* | 113 | 55.1% |
|  | *Missing* | 14 | 6.8% |

Note: Frequency is defined as the total number of patients in the study cohort identified to have the reported variable. ‘Missing’ was used when information about the variable for a patient was unable to be obtained.

**Supplemental Table 2: Tumor variables for entire study cohort**

|  | **Factor/Category** | **Frequency** | **Percent** |
| --- | --- | --- | --- |
| Tumor Differentiation | *Highly Differentiated* | 9 | 4.4% |
|  | *Moderately* | 71 | 34.6% |
|  | *Poorly* | 50 | 24.4% |
|  | *Undifferentiated* | 1 | 0.5% |
|  | *Missing* | 74 | 36.1% |
| Vascular/perineural invasion | *Yes* | 145 | 70.7% |
|  | *No* | 41 | 20% |
|  | *Missing* | 19 | 9.3% |
| Tumor margin | *Yes* | 63 | 30.7% |
|  | *No* | 41 | 20% |
|  | *Missing* | 101 | 49.3% |
| Lymph node status | *Unknown/Missing* | 100 | 48.8% |
|  | *0* | 31 | 15.1% |
|  | *1-3* | 44 | 21.5% |
|  | *>4* | 30 | 14.6% |
| Tumor Location | *Head* | 128 | 62.4% |
|  | *Body and Tail* | 72 | 35.1% |
|  | *Diffuse* | 3 | 1.5% |
|  | *Missing* | 2 | 1% |
| Resectability | *Resectable* | 35 | 17.1% |
|  | *Locally Advanced* | 52 | 25.4% |
|  | *Metastatic* | 116 | 56.6% |
|  | *Missing* | 2 | 1% |
| Number of Metastatic Organs | *1* | 105 | 51.2% |
|  | *2* | 72 | 35.1% |
|  | *3* | 23 | 11.2% |
|  | *≥4* | 5 | 2.4% |
| Size Pulmonary Metastasis | *0-5* | 65 | 31.7% |
|  | *≥6* | 82 | 40% |
|  | *Missing* | 58 | 28.3% |
| Histology | pancreatic ductal adenocarcinoma | 205 | 100% |
|  | IPMN | 2 | 1% |
|  | Neuroendocrine | 1 | 0.5% |

Note: Frequency is defined as the total number of patients in the study cohort identified to have the reported variable. ‘Missing’ was used when information about the variable for a patient was unable to be obtained.

**Supplemental Table 3: Cancer statistics for entire study cohort.**

|  | **Factor/Category** | **Frequency** | **Percent** |
| --- | --- | --- | --- |
| Stage | *1* | 12 | 5.9% |
|  | *2* | 44 | 21.5% |
|  | *3* | 23 | 11.2% |
|  | *4* | 113 | 45.1% |
|  | missing | 13 | 6.3% |
| Initial Surgery | *Yes* | 43 | 21% |
|  | *No* | 159 | 77.6% |
|  | *Missing* | 3 | 1.5% |
| Initial Radiation | *Yes* | 46 | 22.4% |
|  | *No* | 154 | 75.1% |
|  | *Missing* | 5 | 2.4% |
| Recurrent disease Radiation therapy | *Yes* | 34 | 16.6% |
|  | *No* | 139 | 67.8% |
|  | *Missing* | 32 | 15.6% |
| Recurrent Disease Surgery | *Yes* | 5 | 2.4% |
|  | *No* | 171 | 83.4% |
|  | *Missing* | 29 | 14.1% |
| Recurrent Disease Adjuvant Therapy | *Yes* | 32 | 15.6% |
|  | *No* | 133 | 64.9% |
|  | *Missing* | 40 | 19.5% |
| Survival status | *Yes* | 34 | 16.6% |
|  | *No* | 171 | 83.4% |
| Recurrence/Progression | *Yes* | 140 | 68.3% |
|  | *No* | 65 | 31.7% |
| Lung Involvement | *No Lung or Liver* | 34 | 16.6% |
|  | *Lung Only* | 32 | 16.6% |
|  | *Liver and Lung* | 139 | 67.8% |

Note: Frequency is defined as the total number of patients in the study cohort identified to have the reported variable. ‘Missing’ was used when information about the variable for a patient was unable to be obtained.

**Supplemental Table 4: Site of metastasis at diagnosis and site of recurrence or metastasis at time progression.**

| **Site of metastasis at Diagnosis** | **Frequency** | **Percent** |
| --- | --- | --- |
| Liver | 100 | 48.8% |
| Lung | 35 | 17.1% |
| Kidney | 2 | 1.0% |
| Stomach | 7 | 3.4% |
| Spleen | 4 | 2.0% |
| Uterus | 2 | 1.0% |
| Brain | 1 | 0.5% |
| Colon | 4 | 2.0% |
| Other | 35 | 17.1% |
| None | 68 | 33.2% |
| **Site of Recurrence or progression** |  |  |
| Liver | 81 | 39.5% |
| Lung | 54 | 26.3% |
| Kidney | 4 | 2.0% |
| Stomach | 3 | 1.0% |
| Spleen | 2 | 0.7% |
| Uterus | 1 | 0.5% |
| Brain | 2 | 1.0% |
| Colon | 4 | 2.0% |
| Other | 75 | 36.6% |
| Yes | 39 | 19.0% |

Note: Frequency is defined as the total number of patients with reported location of metastasis or recurrence. There were 39 patients identified to have recurrence or progression, however, information about the specific site of recurrence or progression was not available. These patients were categorized into the ‘Yes’ variable reported above.

**Supplemental Table 5: Chemotherapy received.**

| **Initial Chemo Regimen (1^st^ line)** | **Frequency** | **Percent** |
| --- | --- | --- |
| Capecitabine | 17 | 8.3% |
| Gemcitabine | 140 | 68.3% |
| Fluorouracil | 23 | 11.2% |
| Folinic Acid | 20 | 9.8% |
| Cetuximab | 1 | 0.5% |
| Paclitaxel | 96 | 46.8% |
| Irinotecan | 20 | 9.8% |
| Oxaliplatin | 21 | 10.2% |
| Other | 15 | 7.3% |
| Yes | 36 | 17.6% |
| **Recurrent or Progressive Disease Chemo Regimen (2^nd^ and 3^rd^ line)** | **Frequency** | **Percent** |
| Capecitabine | 13 | 6.3% |
| Gemcitabine | 59 | 28.8% |
| Fluorouracil | 40 | 19.5% |
| Folinic Acid | 16 | 7.8% |
| Docetaxel | 1 | 0.5% |
| Cisplatin | 2 |  |
| Paclitaxel | 45 | 22.0% |
| Irinotecan | 35 | 17.1% |
| Oxaliplatin | 20 | 9.8% |
| Other | 17 | 8.3% |
| Yes | 67 | 32.7% |

Note: Frequency is defined as the total number of patients identified to receive reported chemotherapy. The ‘Yes’ variable includes patients known to have received 1^st^, 2^nd^ or 3^rd^ line systemic chemotherapy, but for whom information about specific chemotherapy received was not available.

**Supplemental Table 6: Overall survival for the recurrence free survival and progression free survival cohorts of the IL, LL and NLL groups.**

| **Recurrence Free Survival** |  | N | Events | Median (days) | 95%CI |
| --- | --- | --- | --- | --- | --- |
|  | NLL | 10 | 5 | 1091 | 514, NA |
|  | IL | 10 | 7 | 1650 | 1301, NA |
|  | LL | 18 | 12 | 1503 | 903, NA |
| **Progression Free Survival** |  |  |  |  |  |
|  | NLL | 24 | 21 | 304 | 255, 513 |
|  | IL | 22 | 20 | 501 | 330, 804 |
|  | LL | 121 | 106 | 275 | 225, 406 |

Note: Recurrence Free Survival: N=38, number of events = 24 with event defined as death. Progression Free Survival: N=167, number of events = 147 with event defined as death. Median and Confidence Intervals (CI) are reported in days. ‘NA’ in the confidence intervals were written when there were not enough people who had the corresponding estimate the upper limit of the confidence interval.

**Supplemental Table 7: Survival analysis for overall survival of the recurrence free survival and progression free survival cohorts amongst all three groups (IL, LL and NLL).**

| **Recurrence Free Survival** |  | HR | 95%CI | p-value |
| --- | --- | --- | --- | --- |
|  | NLL | *Ref* | *Ref* | *Ref* |
|  | IL | 0.82 | (0.26, 2.60) | 0.731 |
|  | LL | 1.04 | (0.36,2.99) | 0.942 |
| **Progression Free Survival** |  |  |  |  |
|  | NLL | *Ref* | *Ref* | *Ref* |
|  | IL | 0.73 | (0.39, 1.35) | 0.317 |
|  | LL | 0.97 | (0.61, 1.56) | 0.904 |

Note: CI = confidence interval, HR = hazard ratio. HR greater than 1 would denote a greater probability of the corresponding event and less than 1 would denote a lower probability of the corresponding event (death, recurrence or progression).

**Supplemental Table 8: Survival analysis for overall survival in the recurrence free survival and progression cohorts with only IL and LL groups.**

| **Recurrence Free Survival** |  | HR | 95%CI | p-value |
| --- | --- | --- | --- | --- |
|  | IL | *Ref* | *Ref* | *Ref* |
|  | LL | 1.30 | 0.51, 3.34 | 0.584 |
| **Progression Free Survival** |  |  |  |  |
|  | IL | *Ref* | *Ref* | *Ref* |
|  | LL | 1.32 | 0.82, 2.14 | 0.253 |

Note: CI = confidence interval, HR = hazard ratio. HR greater than 1 would denote a greater probability of the corresponding event and less than 1 would denote a lower probability of the corresponding event (death, recurrence or progression).

**Supplemental Figure 1: Kaplan-Meier Curves for overall survival in the recurrence free survival cohort**


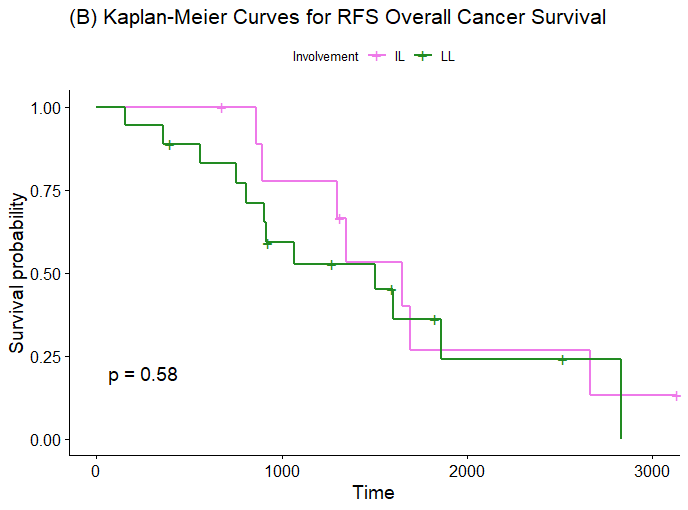

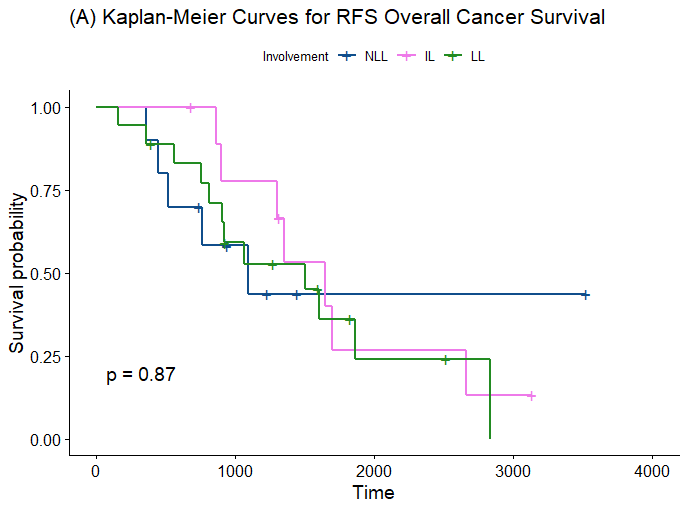


Note: Kaplan-Meier Survival Curves for overall survival in the recurrence free survival cohort for (A) all three groups and (B) when comparing only IL to LL.

**Supplemental Figure 2: Kaplan-Meier Curves for recurrence free survival**


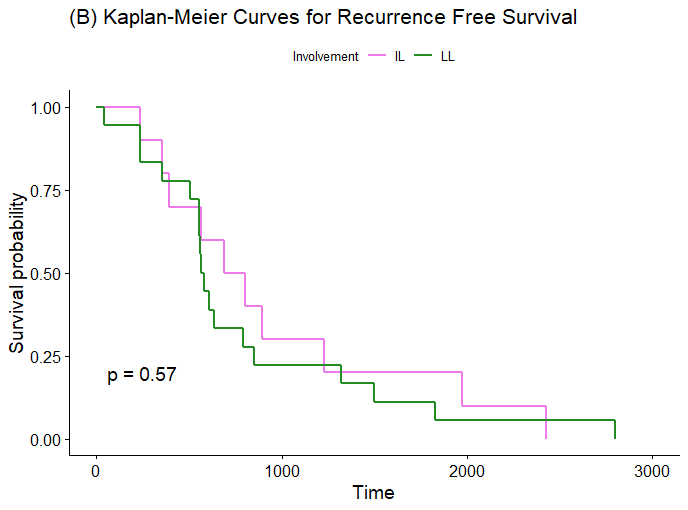

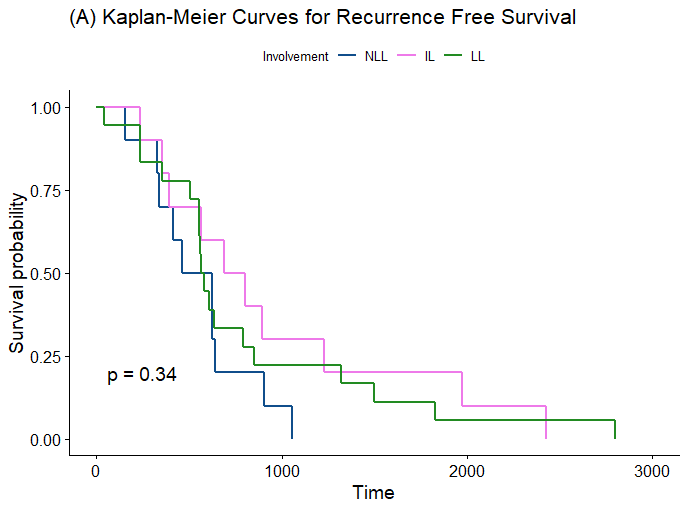


Note: Kaplan-Meier Survival Curves for recurrence free survival for (A) all three groups and (B) when comparing only IL to LL.

**Supplemental Figure 3: Kaplan-Meier Curves for overall survival in the progression free survival cohort**


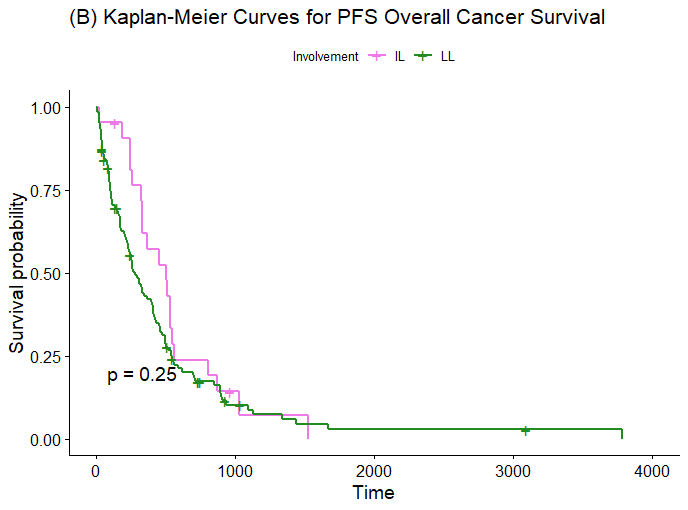

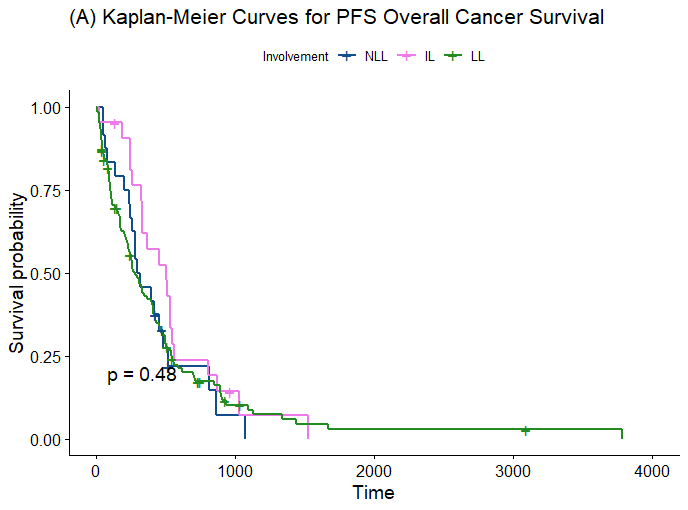


Note: Kaplan-Meier Survival Curves for overall survival in the progression free survival cohort for (A) all three groups and (B) when comparing only IL to LL.

**Supplemental Figure 4: Kaplan-Meier Curves for progression free survival cohort**


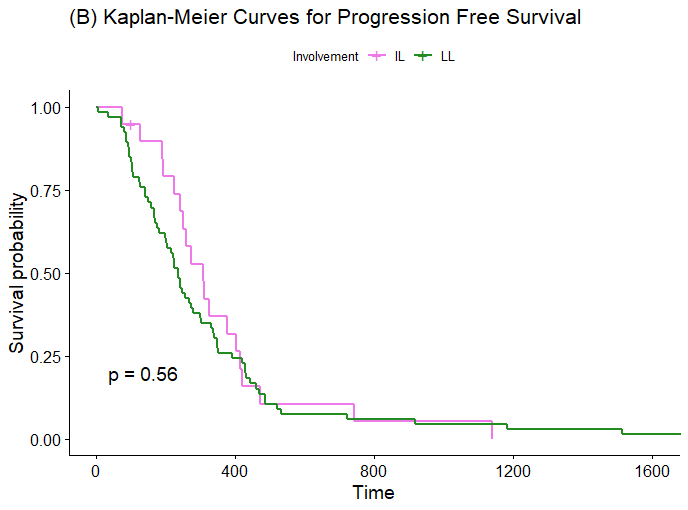

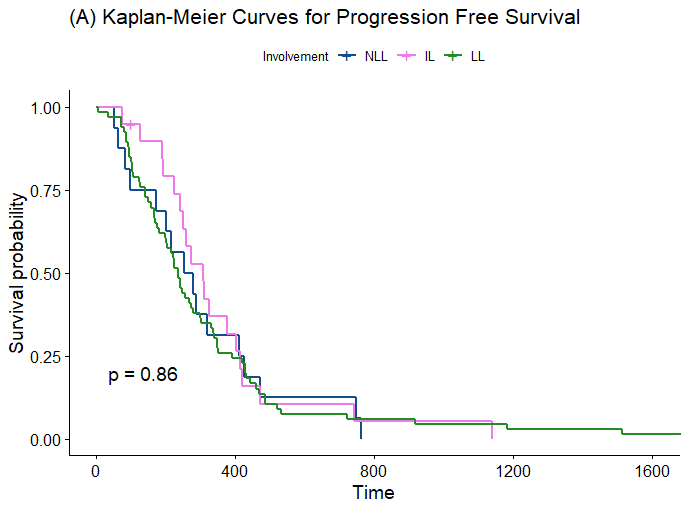


Note: Kaplan-Meier Survival Curves for progression free survival for (A) all three groups and (B) when comparing only IL to LL.
